# Message from the Editor-in-Chief

**DOI:** 10.1155/IJBI/2006/81409

**Published:** 2006-01-30

**Authors:** Ge Wang

**Affiliations:** Department of Radiology, University of Iowa, Iowa City, IA 52242, USA


Hindawi Publishing Corporation
International Journal of Biomedical Imaging
Volume 2006, Article ID 81409, Pages 1–2
DOI 10.1155/IJBI/2006/81409

*Editorial*

### Message from the Editor-in-Chief

**Ge Wang**

*Department of Radiology, University of Iowa, Iowa City, IA 52242, USA*

Received 15 December 2005; Accepted 15 December 2005

Copyright © 2006 Ge Wang. This is an open access article distributed under the Creative Commons Attribution License, which permits unrestricted use, distribution, and reproduction in any medium, provided the original work is properly cited.

The last quarter century has witnessed major advancements that have brought biomedical imaging to a paramount status in the life sciences. As a prominent example, the National Institute for Biomedical Imaging and Bioengineering (NIBIB) was established in 2000 as the newest institute in the National Institutes of Health (NIH), USA. Generally speaking, the scope of biomedical imaging covers data acquisition, image reconstruction, and image analysis, involving theories, methods, systems, and applications. While tomographic and postprocessing techniques become increasingly sophisticated, traditional and emerging modalities play more and more critical roles in anatomical, functional, cellular, and molecular imaging. The overall goal of the International Journal of Biomedical Imaging (IJBI) is to promote research and development of biomedical imaging by publishing high-quality peer-reviewed papers, reviews, and tutorials in this rapidly growing interdisciplinary field.

With the development of the field of biomedical imaging there are many reputable imaging meetings, including the IEEE and SPIE conferences. As a result, the number of high-quality conference papers is well beyond what the current imaging journals handle. In addition, there are new imaging areas that are not specifically targeted by the existing journals. In this context, it is our privilege to launch this new journal. Here I would like to articulate its unique features that justify our efforts, and promise its great potential.

First, this journal will be published using an Open Access publishing model, which means that accepted papers will be freely and immediately available on the journal's website without any access barriers. In addition, a print edition will be made available at a minimal cost. It is our belief that the Open Access model will become a major force in scientific publishing in the near future, and we hope that IJBI will be a leading journal in this new movement.

Second, this journal will promote both theoretical work and innovative techniques and applications. We value the importance of applied mathematics for biomedical imaging, so papers related to this area of research will be quite welcome. Applied mathematics is not only the use of existing mathematical tools in various applications, but also the formulation of mathematical challenges in contemporary research undertakings, as well as the development of new theories, methods, and techniques. In July 2005, Barni and Perez-Gonzalez proposed to push science into signal processing via rigorous experiments with refutation criteria [1]. Their arguments are quite applicable to the development of biomedical imaging. Furthermore, we believe that the status of biomedical imaging can be elevated to a fundamental level using the axiomatic approach, such as the studies on image resolution characterization [2–4]. Guided by the NIH roadmap charted by Zerhouni and many other leading scientists, molecular and cellular imaging research has been significantly boosted over the past years [5–8]. It is foreseeable that increasingly small electromechanical devices, such as quantum dots and nanosensors, may eventually approach the limits to demonstrate quantum behaviors [9]. Quantum imaging may play a significant role in the future of biomedical imaging [10, 11]. Image agents, including contrast materials, molecular probes, and other mechanisms, is critical in the imaging technology. A major direction is to apply sophisticated imaging techniques in biomedical applications. Hence, we welcome all relevant papers from diverse disciplines and fields. We hope to promote the interdisciplinary associations that may lead to research tools and healthcare innovations.

Third, in both the review process and the production process we will aim for the highest possible quality and speed. We have already assembled a first-rate Editorial Board, and we plan to continuously strengthen it in terms of academic authority, specialty balance, geographic scope, junior involvement, and individual productivity. Each board member will make active contributions and be evaluated yearly with routine adjustment for maximum benefit of the journal. Special Issues will be organized to make well-defined contributions to the literature in key areas of research.

We are confident that our journal will soon establish itself as a reputable vehicle in the field, due to the advantages of its Open Access publishing model, comprehensive coverage, novel features, and high academic standards. Please feel free to contact the Editor-in-Chief if you have any ideas or suggestions.

*Ge Wang*

### REFERENCES

[1] M. Barni and F. Perez-Gonzalez, "Pushing science into signal processing," *IEEE Signal Processing Magazine*, vol. 22, no. 4, pp. 119–120, 2005.

[2] G. Wang and Y. Li, "Axiomatic approach for quantification of image resolution," *IEEE Signal Processing Letters*, vol. 6, no. 10, pp. 257–258, 1999.

[3] J. F. Meinel Jr., G. Wang, M. Jiang, T. Frei, M. W. Vannier, and E. A. Hoffman, "Spatial variation of resolution and noise in multi-detector row spiral CT," *Academic Radiology*, vol. 10, no. 6, pp. 607–613, 2003.

[4] M. Jiang, G. Wang, and X.-M. Ma, "A general axiomatic system for image resolution quantification," *Journal of Mathematical Analysis and Applications*, vol. 315, no. 2, pp. 462–473, 2006.

[5] E. Zerhouni, "Medicine. The NIH roadmap," *Science*, vol. 302, no. 5642, pp. 63–72, 2003.

[6] G. Wang, R. Jaszczak, and J. P. Basilion, "Guest editorial toward molecular imaging," *IEEE Transactions on Medical Imaging*, vol. 24, no. 7, pp. 829–831, 2005.

[7] V. Ntziachristos, J. Ripoll, L. V. Wang, and R. Weissleder, "Looking and listening to light: the evolution of whole-body photonic imaging," *Nature Biotechnology*, vol. 23, no. 12, pp. 313–320, 2005, (with a section "Bioluminescence tomography" commenting on our molecular imaging work).

[8] G. Wang, Y. Li, and M. Jiang, "Uniqueness theorems in bioluminescence tomography," *Medical Physics*, vol. 31, no. 8, pp. 2289–2299, 2004.

[9] K. C. Schwab and M. L. Roukes, "Putting mechanics into quantum mechanics," *Physics Today*, vol. 58, no. 7, pp. 36–42, 2005.

[10] R. P. Feynman, R. B. Leighton, and M. Sands, *The Feynman Lectures on Physics. Vol. 3*, Addison-Wesley, Reading, Mass, USA, 1965.

[11] Y. Shih, "Entangled photons," *IEEE Journal of Selected Topics in Quantum Electronics*, vol. 9, no. 6, pp. 1455–1467, 2003.

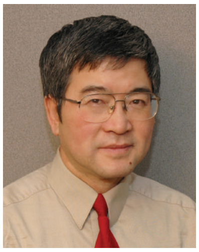

**Ge Wang** received his B.E. degree in electrical engineering from Xidian University, Xi'an, China, in 1982, his M.S. degree in remote sensing from Graduate School of Academia Sinica, Beijing, China, in 1985, and his M.S. and Ph.D. degree in electrical and computer engineering from State University of New York, Buffalo, in 1991 and 1992. He was an Instructor and Assistant Professor with the Department of Electrical Engineering, Graduate School of Academia Sinica in 1984–1988, Instructor and Assistant Professor with Mallinckrodt Institute of Radiology, Washington University, St. Louis, Mo, in 1992–1996. He was an Associate Professor with University of Iowa from 1997 to 2002. Currently, he is a Professor with the Departments of Radiology, Biomedical Engineering, Mathematics, Civil Engineering, and Electrical and Computer Engineering, and Director of the Center for X-Ray and Optical Tomography, University of Iowa. His interests include computed tomography, bioluminescence tomography, and systems biomedicine. He has published over 300 journal articles and conference papers, including the first paper on spiral/helical cone-beam CT and the first paper on bioluminescence tomography. He is the Editor-in-Chief of International Journal of Biomedical Imaging and Associate Editor for IEEE Transaction Medical Imaging and Medical Physics. He is an IEEE Fellow and an AIMBE Fellow. He is also recognized by a number of awards for academic achievements.

